# Metabolism, Mitochondrial Dysfunction, and Redox Homeostasis in Pulmonary Hypertension

**DOI:** 10.3390/antiox11020428

**Published:** 2022-02-21

**Authors:** Daniel Colon Hidalgo, Hanan Elajaili, Hagir Suliman, Marjorie Patricia George, Cassidy Delaney, Eva Nozik

**Affiliations:** 1 Division of Pulmonary Sciences and Critical Care Medicine, University of Colorado Anschutz Medical Campus, Aurora, CO 80045, USA; daniel.colonhidalgo@cuanschutz.edu; 2 Division of Pediatrics-Critical Care, University of Colorado Anschutz Medical Campus, Aurora, CO 80045, USA; hanan.elajaili@cuanschutz.edu; 3 Department of Anesthesiology, Duke University Medical Center, Durham, NC 27710, USA; hagir.suliman@duke.edu; 4 Division of Pulmonary, Critical Care & Sleep Medicine, National Jewish Health, Denver, CO 80206, USA; georgem@njhealth.org; 5 Section of Neonatology, University of Colorado Anschutz Medical Campus, Aurora, CO 80045, USA; cassidy.delaney@childrenscolorado.org

**Keywords:** pulmonary hypertension, glycolytic switch, ROS, mitochondria

## Abstract

Pulmonary hypertension (PH) represents a group of disorders characterized by elevated mean pulmonary artery (PA) pressure, progressive right ventricular failure, and often death. Some of the hallmarks of pulmonary hypertension include endothelial dysfunction, intimal and medial proliferation, vasoconstriction, inflammatory infiltration, and in situ thrombosis. The vascular remodeling seen in pulmonary hypertension has been previously linked to the hyperproliferation of PA smooth muscle cells. This excess proliferation of PA smooth muscle cells has recently been associated with changes in metabolism and mitochondrial biology, including changes in glycolysis, redox homeostasis, and mitochondrial quality control. In this review, we summarize the molecular mechanisms that have been reported to contribute to mitochondrial dysfunction, metabolic changes, and redox biology in PH.

## 1. Introduction

Pulmonary hypertension (PH) represents a myriad of disorders characterized by elevated mean pulmonary artery pressure. It is a progressive and, often, a lethal disease in both children and adults. Pulmonary hypertension is divided into five clinical classifications or groups, depending on the underlying etiology [1,2]. Pulmonary vasoconstriction and vascular remodeling, to varying degrees, are commonly encountered in group I pulmonary arterial hypertension (PAH) [3]. Standard treatment targets different pathways to augment vasodilation [4]. Despite these therapeutic interventions, the prognosis is generally poor, necessitating further investigation into the pathogenesis of the aforementioned vascular remodeling to improve novel therapeutic strategies [5]. Vascular remodeling is, in part, attributable to resistance to apoptosis and uncontrolled proliferation of the vascular resident cells, such as endothelial and smooth muscle cells, as well as matrix deposition [6]. One emerging factor contributing to pulmonary vascular remodeling is mitochondrial dysfunction and metabolic disturbances [7]. This review aims to summarizes the current understanding of the role of mitochondrial dysfunction in the development of PH. This will include electron transport chain (ETC) dysfunction and the shift in energy production from mitochondrial oxidative phosphorylation to glycolysis, mitochondrial DNA damage, impaired quality control (biogenesis and mitophagy), imbalances in mitochondrial dynamics (fission and fusion), mitochondrial membrane hyperpolarization, and the altered production of ROS (Table S1). A cutting-edge understanding of the mitochondrial metabolic, molecular, and physiologic role in this disease should enable the development of mitochondria-targeted therapies to slow or revert PH development.

## 2. Glycolytic Switch and Energy Source in Pulmonary Hypertension

Resistance to apoptosis and the unregulated hyperproliferation of pulmonary artery smooth muscle cells (PASMCs) contribute to pulmonary vascular remodeling [8]. This remodeling, stemming from the increased survival and proliferation of PASMCs, has been linked with changes in mitochondrial metabolism, more specifically, a transition from oxidative phosphorylation to glycolysis, a process also referred to as the Warburg effect, or glycolytic switch [9]. The Warburg effect has been well described in tumorigenesis, providing energy and substates to enable the rapid growth of tumor cells [10]. Several possible advantages to the glycolytic switch have been postulated. First, there is an acceleration in the glycolytic rate and turnover of glucose into lactic acid, resulting in faster, yet still low-yield, adenosine triphosphate (ATP) production [11,12]. In addition to ATP production, glycolysis allows for the formation of important intermediate molecules that are critical for the synthesis of macromolecules and the promotion of the pentose phosphate pathway (PPP) and therefore, cell proliferation. Lastly, the increased production of NADPH in glycolysis results in increased glutathione, which has been associated with chemoresistance through its interaction with drugs, the prevention of DNA damage, and its reaction with ROS [13,14]. The metabolic changes and glycolytic shift seen in pulmonary hypertension are proposed to be analogous to the Warburg effect in cancer biology [15].

Several critical enzymes within the glycolytic pathway are upregulated and linked to the accelerated growth properties of pulmonary vascular cells in pulmonary hypertension. α-Enolase (ENO1) has been implicated in pulmonary artery smooth muscle cell hyperproliferation in pulmonary hypertension [16]. ENO1 levels are elevated in both animal models of hypoxic pulmonary hypertension, as well as in patients with PAH. The overexpression of ENO1 is associated with an apoptosis-resistant PASMCs phenotype mediated through the AMPK-Akt pathway. The pharmacologic inhibition of ENO1 led to decreases in the glycolytic switch under hypoxic conditions, and pharmacologic inhibition reversed pulmonary hypertension in animal models [16]. In addition to ENO1’s role in the pathogenesis of pulmonary hypertension, upregulation of other key glycolytic enzymes, such as glucose-6-phosphate dehydrogenase (G6PD), hexokinase, pyruvate dehydrogenase, and pyruvate dehydrogenase kinase, have also been implicated in driving pulmonary hypertension [17]. The function of G6PD, the rate-limiting enzyme for the pentose phosphate pathway (PPP), has been linked to PH. When overexpressed, G6PD leads to the activation of the PPP pathway, which is critical for nucleotide synthesis and PASMC cellular proliferation [18]. In hypoxic cells, increased G6PD activity is associated with the upregulation of hypoxic inducible factor α (HIFα), cyclin A, and phosphohistone H3, resulting in dedifferentiation and cell cycle dysregulation [18]. In a PH mouse model, the pharmacological inhibition of G6PD led to decreased RV pressure overload [19]. Paradoxically, G6PD deficiency in mice, though it did not reduce PPP flux, increased oxidative stress and activation to several signaling pathways that contribute to SMC proliferation [20]. Interestingly, a study of twenty-two patients with idiopathic PAH demonstrated moderately decreased G6PD activity in three patients, suggesting this as potential mechanism in selected patients [20,21]. These data suggest that either an increase or decrease in normal G6PD activity can be associated with PH. Hexokinase, one of the limiting enzymes in the glycolytic pathway, has also been studied in PH. The upregulation of hexokinase, assessed via mRNA and protein expression, has been described in rat models of PH, and pharmacologic inhibition is associated with the amelioration of hemodynamic changes and SMC proliferation [22,23]. Increased levels of pyruvate dehydrogenase kinase (PDK), which inhibits pyruvate dehydrogenase (PDH), a key enzyme for glucose oxidation, have been found in the pulmonary arteries of patients with PAH. The treatment of explanted PAH lungs with a PDK inhibitor, dichloroacetate (DCA), activated PDH and increased mitochondrial respiration. Furthermore, an open-label trial of DCA administration to patients with idiopathic PAH showed improvement of hemodynamic parameters in selected patients [24]. Collectively, these studies illustrate that alterations in key glycolytic enzymes mediate the development of pulmonary hypertension.

The changes in glycolysis predilection seen in pulmonary vascular resident cells have also been described in distant/circulating cell types. In skeletal muscle, proteomic and metabolic analysis of the quadriceps muscles of patients with PAH demonstrated a lower expression of pyruvate dehydrogenase (PDH), a key oxidative phosphorylation enzyme, and a higher expression of lactate dehydrogenase, an enzyme associated with anaerobic glycolysis, when compared to healthy controls [25]. The inhibition of PDH by pyruvate dehydrogenase kinase (PDK) also occurs in right ventricle (RV) myocytes, leading to a glycolytic switch that has been associated with a reduction in contractility. The inhibition of PDK (therefore, a re-activation of PDH) led to the increased oxidation of glucose, improvement in RV function, and regression of pulmonary vascular disease [26].

In circulating cells, like platelets, similar changes have been identified. In patients with PAH, platelets were found to have higher basal glycolytic rates and lower reserve glycolysis when compared to control subjects. Moreover, there was a correlation between platelet glycolytic parameters and the severity of PAH [27]. Another study demonstrated that platelets from PAH patients show increased glycolytic rates by extracellular flux, increased maximal capacity for oxygen consumption, and increased respiratory reserve capacity; these changes also correlated with RV function, pulmonary vascular resistance, and pulmonary artery pressures. The increased platelet reserve capacity was found to be due to increases in fatty acid oxidation and was reversed by etomoxir, a pharmacological inhibitor that blocks fatty acid transport into the mitochondrion [27]. This represents an important concept: these circulating cells might exhibit changes similar to those seen in resident cells, suggesting the systemic nature of the metabolic changes seen in PH. Furthermore, the ability to easily monitor this process through the analysis of platelets could potentially allow for the monitoring of therapeutic responses or disease progression.

Elevated glucose uptake and the use of glucose as a predominant metabolic substrate may also play a role in PH. In rats subjected to the Sugen/hypoxia model in order to develop severe PH, there was an increase in glucose usage in the RV measured by nuclear imaging [15,28]. The effects of excess glucose in RV myocytes have also been studied. Increased glucose in the myocytes can enter the hexosamine biosynthetic pathway (HBP), leading to the conversion of glucose into uridine-disphosphate-N-acetylgyclosamine, which results in the reinforcement of metabolic abnormalities and mitochondrial dysfunction via the GlcNAcylation of mitochondrial proteins. Moreover, colchicine has been found to have a partially beneficial effect in MCT-treated rats via the inhibition of excess O-GlcNAcylation [29]. Overall, these data support the importance of glucose uptake and utilization in PH.

Emerging evidence suggests that fatty acid (FA) uptake, oxidation, and utilization are disrupted in PH. In rapidly dividing cells, increased fatty acid oxidation (FAO) has been associated with survival [30]. In addition, rapidly dividing cells have been shown to have higher de novo fatty acid synthesis rates [31]. In PH, the inhibition of FAO by targeting malonyl CoA decarboxylase, or 3-ketoacyl CoA, was found to be protective [32]. In a rat model of PH and human pulmonary artery smooth muscle cells (HPASMC) subjected to hypoxia, the inhibition of fatty acid synthase (FAS) by siRNA led to increases in apoptosis and glucose oxidation while the mitochondrial in HPASMC demonstrated normal ROS levels and membrane potential. In rats, these metabolic changes due to FAS inhibition correlated with decreased RV pressure, hypertrophy, and pulmonary vascular remodeling [33]. Interestingly, RV myocytes have different patterns of substrate use. Sakao et al. performed metabolomics on the RV of rats subjected to Sugen/hypoxia to develop PH [34]. They reported a decrease in malic and fumaric acid when compared to controls. In addition to this, long-chain acylcarnitines, which are important for FA transport to the mitochondria, were reduced in pulmonary hypertension, suggestive of decreases in FA oxidation [34]. Studies in human PAH have also demonstrated these changes in substrate predilection in the RV, correlating with the severity of the disease, as well as the ability to detect abnormalities in FA metabolism from the serum of patients with PAH [35,36,37]. This discordance between pulmonary artery smooth muscle cell and RV fatty acid oxidation could contribute to the underwhelming results of metabolic therapy in pulmonary hypertension [38].

## 3. Mitochondrial Quality Control

The processes responsible for mitochondrial quality control ensure healthy mitochondrial function and cellular bioenergetics. These processes include proteostasis, biogenesis, dynamics (fission and fusion), and mitophagy [39]. Proteostasis refers to maintaining normal protein folding and structure, which is key for normal protein localization and function [40]. Biogenesis pertains to the control of mitochondrial growth and division [41]. Fission, the process of one mitochondrion splitting into two, and fusion, the joining of two mitochondria into one, are central to mitochondrial dynamics [42,43]. Lastly, mitophagy refers to the process of mitochondrial removal via autophagy [44]. A range of disruptions in mitochondrial quality control has been seen in PH models.

While cytosolic proteostasis and the unfolded protein response (UPR) to endoplasmic reticulum (ER) stress have been associated with many human diseases [45], mitochondrial proteostasis and its role in human disease remains largely under-investigated. One clue that mitochondrial proteostasis could be important in pulmonary circulation is based on a study in rats treated with antimycin A (AA), a mitochondrial complex III inhibitor. In this study, proteomic analyses of lung tissue showed the malfunction of protein clearance and detoxification, which the authors suggested could account for AA-induced pulmonary vascular constriction [43,46]. Despite limited information on the role of mitochondrial proteostasis in PH, there is more evidence supporting a role in cardiac disease. For example, in a model of cardiac ischemia-reperfusion injury, the upregulation of LonP1, a mitochondrial protease key in mitochondrial proteostasis, was shown to be protective, demonstrated by a significantly smaller infarct territory and decreased apoptosis [47]. Given its role in ischemic heart disease, it is plausible that mitochondrial proteostasis could also contribute to the development of right ventricular dysfunction in PH.

Accumulating studies support the idea that impaired mitochondrial biogenesis also plays a role in PH pathogenesis. As a first line of evidence, PAH PASMCs have a lower expression of voltage-dependent anion-selective channels (VDAC) and citrate synthase, both markers of mitochondrial mass [48,49]. In addition, key mitochondrial transcription factors and co-regulators are disrupted in pulmonary hypertension. Mitochondrial biogenesis is controlled by PGC-1α [PPAR (peroxisome proliferator-activated receptor)-γ coactivator 1α], a co-transcriptional regulator that promotes mitochondrial biogenesis by enhancing the activation of nuclear respiratory factors 1 and 2 (NRF1 and NFR22) and peroxisome proliferator-activated receptor-γ (PPARγ). NFR1, NFR2, and PPARγ activation promote mitochondrial transcription factor A (Tfam) and mitochondrial DNA (mtDNA) replication and transcription [41]. Exposure to hypoxia leads to a decrease in the expression of PPARγ, with a resulting lower expression of PGC-1α, a decline in the expression of Tfam, VDAC, mitofusin-2 (MFN2), and heat shock protein family A member 9 (HSPA9), and a dysregulation of mitochondrial biogenesis [50]. The pharmacologic antagonism of PPARγ, or the administration of siRNA against PPARγ or PGC-1α, increased cell proliferation, decreased mitochondrial mass, and decreased mitochondrial biogenesis, while hypoxia-induced changes in mitochondrial biogenesis were reversed by PGC-1α overexpression in HPASMCs, overall supporting the role of these pathways in PH pathophysiology [50]. In a pulmonary hypertension rat model, the administration of pioglitazone, a PPARγ agonist, decreased RVSP and prevented RV dilation, which could in part be due to its impact on mitochondrial biogenesis [51]. Unfortunately, there remains a paucity of evidence regarding the role of PPARγ in patients with pulmonary hypertension. Sirtuin 1 (SIRT1), an NAD^+^-dependent deacetylase, exerts another level of control of mitochondrial transcription by deacetylating and activating PGC-1α [52]. SIRT1 knockdown rats demonstrated higher vascular remodeling when exposed to hypoxia, whereas SIRT1 activation inhibited both rat and human PASMC proliferation and led to a higher expression of mitochondrial biogenesis markers [52]. Lastly, nitric oxide (NO), a known vasodilator, plays an integral part in the control of cellular respiration and mitochondrial biogenesis [53]. In a fetal model of persistent PH of the newborn (PPHN), decreased NO production related to reduced expression of nitric oxide synthase led to impairments of mitochondrial biogenesis. In this model, these impairments included decreased levels of PGC-1α, mtDNA, and ETC complex expression, which were partially reversed by NO donor administration [54].

Mitochondrial dynamics, via fission and fusion, are crucial for the maintenance of mitochondrial distribution, shape, and size, which in turn are key in different processes such as mitochondrial quality control, and therefore functions such as cell cycle, proliferation, and apoptosis [55]. The disruption of normal mitochondrial dynamics has been associated with several different human diseases [56]. Enzymes such as dynamic-related protein 1 (DRP1) and dynamin2 (DNM2) play an important role in the regulation of fission [57]. DRP1 translocates to the outer mitochondrial membrane (OMM) and binds to receptors such as Fis1, MiD49/51, and Mff and then forms ring-shaped oligomers that lead to the formation of the “constriction point” in the mitochondria, essential for fission [58,59]. Mitochondrial fragmentation has been previously observed in PAH PASMCs; in human PASMCs, DRP-1 was found to be crucial for cell-cycle checkpoints, and its overexpression was hypothesized to contribute to hyperproliferation [60,61]. In a rat model of PAH, right ventricular fibroblasts showed an increased expression of DRP1, and its inhibition led to reductions in right ventricular fibroblast proliferation and the production of collagen [62]. Treprostinil, a commonly used prostacyclin analog in patients with PAH [63,64], has been shown to stimulate the phosphorylation of DRP1 via protein kinase A (PKA), resulting in the inhibition of DRP1 and thus, increases in mitochondrial fusion and elongation in PASMCs [65,66]. High-mobility group box-1 (HMGB1) has been previously identified as a biomarker of PAH and correlates with the severity of disease. The inhibition of HMGB1 has been associated with decreased pulmonary vascular remodeling in different pulmonary hypertension rat models. Recently, investigators found that HMGB1, released from damaged cells, leads to the phosphorylation of DRP1 and fission through the activation of the ERK1/2 pathway and autophagy activation. The treatment of rats with an inhibitor of HMGB1/TLR4 interaction has demonstrated therapeutic effects [66,67]. Mitochondrial fusion is regulated by mitofusin 1 (MFN1) and mitofusin 2 (MFN2), mediators of outer mitochondrial membrane fusion, and optic atrophy 1 (OPA1) mediates inner mitochondrial membrane fusion. Mitochondrial fusion, like fission, is an important influencer of cellular proliferation. For example, when cloned, MFN2 was found to have an antiproliferative effect [68,69]. In normal HPASMCs, the reduction of MFN2 expression led to increased mitochondrial fragmentation and increased cellular proliferation. In addition, in HPASMCs from PAH patients, MFN2 was decreased, and there was a higher incidence of mitochondrial fragmentation. In rodent and human PAH PASMCs, adenoviral-mitofusin-2 (Ad-MFN2) overexpression led to a decrease in pulmonary vascular resistance and PA medial thickness, and an increase in lung vascularity [70]. There are several different possible mechanisms leading to decreased MFN2 expression in pulmonary hypertension. The lower expression of PGC-1α, a transcriptional cofactor in the MFN2 promoter, leads to lower MFN2 transcription. Secondly, the estrogen-related receptor-alpha (ERR-α) is another transcriptional cofactor in the MFN2 promoter, which also binds PGC-1α for activation; this latter mechanism could be partly responsible for the increased PAH in females [71,72]. Lastly, in systemic vascular SMCs, stimuli such as endothelin-1 and platelet-derived growth factor (PDGF) are known to downregulate MFN2 [73,74]. The role of MFN1 in PH has not been fully described, but it has been previously tied to the regulation of lipid metabolism in a model of pulmonary fibrosis, providing a further rationale for determining its role in PH [75]. In addition, the activation of OPA1 via the administration of BGP-15 (O-[3-piperidino-2-hydroxy-1-propyl]-nicotinic amidoxime) has been shown to promote mitochondrial fusion, protect lung structure, and stabilize cristae membranes in a pulmonary hypertension model [76].

Mitophagy, a key mitochondrial quality-control process, refers to the degradation of excess or damaged mitochondria. It promotes the re-institution of homeostasis in response to cellular stress [77]. Several different stimuli can lead to the activation of mitophagy, such as oxidative stress, starvation, or programmed mitochondrial removal [78,79,80]. Abnormalities in mitophagy have been associated with a multitude of human diseases, along with aging [81,82]. Mitophagy has been previously described as occurring in both a non-selective and selective way. Non-selective (type 1) mitophagy, as the name implies, deals with a general “clearance” of excess mitochondria rather than only damaged ones; this is commonly encountered during nutrient starvation states [83,84]. Selective (types 2 and 3) mitophagy are more specific, as only damaged mitochondria are targeted. Type 2 involves mitochondrial sequestration in the autophagosomes, and type 3 involves vesicles budding off the mitochondria and being trafficked to the lysosomes [85]. Several key pathways have been described in mitophagy. These pathways include PTEN-induced putative kinase 1 (PINK1) and Parkin RBR E3 ubiquitin-protein ligase (PRKN, also known as Parkin) [86,87]. PINK1 accumulates in the outer mitochondrial membrane during mitochondrial stress, which leads to the accumulation and activation, via phosphorylation, of Parkin [88]. Activation of Parkin leads to the downstream ubiquitination of outer mitochondrial proteins such as VDAC and mitochondrial Rho GTPase (MIRO) [89,90]. This outer mitochondrial membrane protein ubiquitination results in the engagement of autophagy receptors, such as calcium binding and coiled-coil domain 2 (CALCOCO2, also known as NDP52), optineurin (OPTN), and Tax1 binding protein (TAX1BP1) [91]. These autophagy receptors then aid in the assembly of different proteins of the autophagocytic machinery. Although its role in PH has not been fully examined, previous investigators have suggested that mitophagy plays a role in the development of PH and other pulmonary diseases such as acute lung injury, idiopathic pulmonary fibrosis, and chronic obstructive pulmonary disease [92]. In PASMCs, cyclosporine, an inhibitor of mitophagy, reduced proliferation when these cells were exposed to hypoxia, suggesting that increased mitophagy is involved in PASMC proliferation [93]. Uncoupling protein 2 (UCP2) is an anion transporter in the inner mitochondrial membrane that regulates mitophagy [94]. UCP2 knockdown mice have been shown to exhibit worse hypoxic pulmonary hypertension, which was thought to be related to ER stress and mitochondrial hyperpolarization in PASMCs [95]. The loss of UCP2 in endothelial cells led to increases in mitophagy, as measured by the PINK1/LC3BII/I ratio, decreased biogenesis, and increased apoptosis [96]. Despite this evidence, other studies suggest that inadequate mitochondrial clearance by mitophagy is seen in the PASMCs of patients with PAH [7,97]. These differing conclusions could represent that rather than increased or decreased mitophagy, an imbalance of mitophagy as a means to regulate mitochondrial is associated with PH development.

## 4. Nuclear and Mitochondrial DNA Damage and Pulmonary Hypertension

The structural integrity of DNA can be damaged by exposure to cellular metabolites and exogenous agents. The DNA sequence can be altered by polymerase disruption during replication or by environmental causes such as mutagenic chemicals, oxidative stress, radiation, and chronic inflammation. These changes can vary from a single base to complex structural changes. Based on the type of DNA damage, relevant DNA damage response (DDR) pathways are stimulated. These responses aim to restore the DNA duplex, prevent transmission of damaged nuclear DNA, and initiate apoptosis signaling if the damage is unrepairable [98].

If these damages are not properly repaired, cells accumulate mutations in their genome, which can lead to death by apoptosis. Dysfunctional nuclear DNA-damage response mechanisms lead to apoptosis-resistant and hyper-proliferative phenotypes implicated in vascular remodeling [99].

In the past decade, several studies implicated genomic instability and increased levels of nuclear DNA damage in PAH lung vascular cells. Moreover, the dysregulated DDR pathways have been identified as the underlying cause for the presence of persistent nuclear DNA damage [100].

Mitochondrial DNA (mtDNA) encodes for critical mitochondrial genes necessary for mitochondrial function. Recent studies reported the observation of DNA damage in nuclear and mitochondrial genomes of PAH patients. Despite a lack of understanding of the events that occur in the early stages of PAH, emerging data suggest that oxidative stress and inflammation, known to impact vascular cell contractility and proliferation, are also linked to nuclear and mitochondrial DNA damage [100].

## 5. ROS Production

Oxidative stress in PH has been previously described; however, the success of antioxidant therapy in animal PH models has not translated to similar efficacy with its use in human disease. One important consideration is that the prior definition of oxidative stress as an imbalance between oxidants and antioxidants is incomplete, with an emerging recognition that reactive oxygen species serve as key signaling molecules. Another key concept is that the site of ROS production is compartmentalized, and specific targeting of mitochondrial ROS, for example, may be a future strategy for improving mitochondrial function and thus, PH. This paradigm shift in the field of redox biology has led to increased research efforts to understand the site-specific redox-regulated processes which are disrupted in disease states and the need to restore homeostasis.

In mammalian cells, the mitochondria represents a principal source of ROS production [101,102]. The electron transport chain (ETC) in the mitochondrial membrane is a key component in ROS production. The ETC is composed of complexes I-IV (CI-CIV) and the electron transfer carriers cytochrome c and ubiquinone, all located in the inner mitochondrial membrane [103,104]. Electrons from NADH are transferred from CI-CIII-CIV or CII-CII-CIV, releasing energy at each transfer. This energy drives the synthesis of adenosine triphosphate (ATP) by ATP synthase (complex V) [104]. Under normal conditions, a small proportion of electrons escape the electron transport chain and interact with oxygen to produce superoxide (O_2_^•−^). Superoxide dismutase 2 (SOD2) catalyzes the conversion of O_2_^•−^ into H_2_O_2_ in the inner mitochondrial membrane [105,106]. The role of mitochondrial ROS in PH has been extensively studied, but it remains a subject of controversy. In models of chronic hypoxic PH, previous studies have shown that hypoxia leads to an increase in the mitochondrial production of superoxide in PASMCs [107,108,109]. In a pulmonary hypertension rat model, PASMCs demonstrated lower activities in CI-CIII, which was then associated with an increase in ROS generation [110]. In a PH rat model, PASMCs demonstrated lower activities in CI-CIII, which was then associated with an increase in ROS generation [110]. In addition, mutations like NFU1, a mitochondrial scaffolding protein important in the biosynthesis of iron-sulfur clusters, have also been associated with sporadic cases of PH, among other abnormalities [111,112]. NFU1 mutations are thought to contribute to the dysregulation of ROS homeostasis in the mitochondria, leading to increased ROS production, resistance to apoptosis, and increased proliferation in PASMCs [113]. Rats with the G208C mutation (a human mutation) of the NFU1 were found to have increased right ventricular pressure, right ventricular hypertrophy, and pulmonary artery remodeling, including angioobliterative changes [114]. Despite the seemingly compelling evidence that increased ROS production is associated with PH, other authors have reported contradictory findings. In both rat and human-derived pulmonary artery smooth muscle cells, reductions in the expression of electron transport chain components and superoxide dismutase-2 were found, leading to decreased ROS production and therefore, the activation of HIF-1α in normoxia and disruptions of oxygen sensing mechanisms. These changes were thought to lead to similar pathophysiologic changes in chronic hypoxia [115]. These abnormalities were pharmacologically targeted, leading to improvement in mitochondrial function and causing regression of the disease [115,116,117]. These differences in conclusions regarding the role of ROS in PH pathophysiology have been hypothesized to be due to several factors, including the use of different models (experimental conditions and species studied), as well as the complexity of in vivo ROS measurements and need for further understanding of the importance of the timing and location of ROS production [118].

Given the previously described role of ROS in pulmonary hypertension, targeting mitochondrial ROS as a therapeutic intervention has also been studied. For example, hypoxia-exposed mice treated with a mitochondrial-specific antioxidant, MitoQ, were found to have a decrease in hypoxic pulmonary vasoconstriction and a decrease in the rise of superoxide concentration after an exposure to acute hypoxia. In chronic hypoxia, however, MitoQ did not influence the development of pulmonary hypertension, but it did reduce RV remodeling [117]. These results suggest that the timing of mitochondrial ROS production is key in the development of PH. MitoTEMPO is a mitochondrial-specific superoxide dismutase (SOD) mimetic that has previously been studied for use in cases of vascular oxidative stress, demonstrating vascular relaxation and a decrease in systemic blood pressure after mice were exposed to angiotensin II (to induce systemic hypertension) [119]. The role of mitochondrial SOD-mimetic therapy in pulmonary hypertension remains to be investigated.

In addition to mitochondrial ROS production, the extracellular redox environment may also play a role in mitochondrial biology and PH pathogenesis. Extracellular superoxide dismutase (ECSOD or SOD3), essential for extracellular ROS homeostasis, has also been closely linked to the development of pulmonary hypertension. SOD3 whole-body knockdown, SMC deletion, and a single nucleotide polymorphism (SNP) in the matrix binding region have all been associated with more severe hypoxic PH in mice [120,121,122]. In adults with PH, there was a correlation between lower SOD3 activity, pulmonary vascular resistance (PVR), and mortality [123]. The interaction between intracellular and extracellular ROS homeostasis in pulmonary hypertension remains to be fully elucidated, but this could be a potential area of further investigation regarding the pathogenesis of pulmonary hypertension. Interestingly, a recent study showed a link between SOD3 and glucose metabolism via the activation of the AMPK pathway in a fetal liver; this hints to a connection between extracellular ROS homeostasis and glycolysis [124]. In PH, the effect of extracellular redox potential and mitochondrial quality control and the resulting influence on mitochondrial ROS production is an area that is yet to be fully explored.

## 6. Apoptosis Resistance

Apoptosis resistance in pulmonary vascular cells is one of the hallmarks of pulmonary hypertension. Interestingly enough, some of the mitochondrial processes previously mentioned contribute to this phenotype. The dysregulation of redox homeostasis leads to mitochondrial dysfunction and mitophagy, resulting in lower mitochondrial mass. This further impairs ATP production and promotes glycolysis to increase the substrates necessary for cell proliferation. In addition, increased glycolytic rates lead to hyperpolarization of the inner mitochondrial membrane, halting pro-apoptotic factors [9,92,125]. Lastly, in a rat model of PH and in patients with PAH, the release and accumulation of cytosolic survivin, a caspase inhibitor, promotes apoptosis resistance [126]. These changes suggest a strong relationship between mitochondrial dysfunction and impaired programmed cell death.

## 7. Conclusions

**Figure 1:**
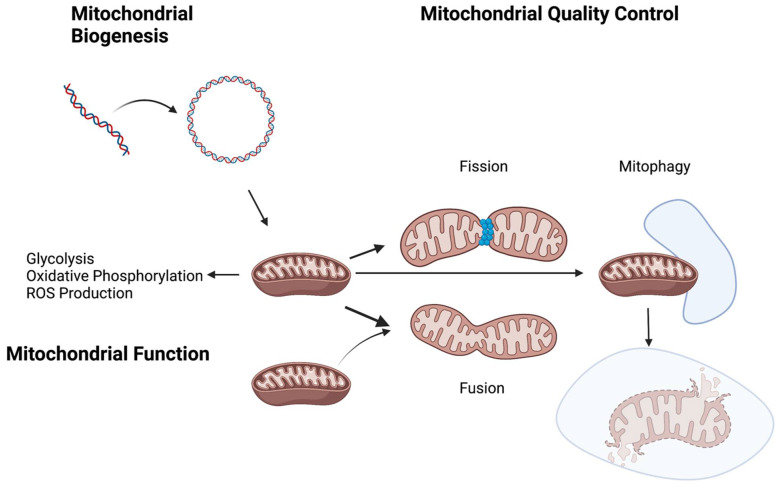
The disruption to several metabolic and/or mitochondrial processes, such as the glycolytic switch and oxidative phosphorylation, as well as changes in mitochondrial biogenesis and quality control, have been associated with the development of pulmonary hypertension. Created with BioRender.com.

In conclusion, there are well-documented changes in the metabolism, ROS homeostasis, and mitochondrial quality control in cell culture, mouse models, and HPASMCs (Figure 1). Similar to cancer biology, the Warburg effect, or glycolytic switch, has been well-described in pulmonary artery smooth muscle cells, as it leads to hyperproliferation. Furthermore, the dysregulation of mitochondrial biogenesis and dynamics have also been previously reported in the literature. Other contributing factors, such as the role of mitophagy and ROS production, remain uncertain, as contradicting results have been described. With the advancement of molecular techniques, it is possible that such controversies might be resolved, and links may be found among the different major molecular abnormalities commonly described in PH. Further studies could help elucidate potential signaling molecules that might be key players in these interactions, thus providing further therapeutic target candidates for patients with PH.

## Supplementary Materials

The following are available online at https://www.mdpi.com/article/10.3390/antiox11020428/s1, Table S1: A summary of the key enzymes of different mitochondrial processes associated with animal models of PH and/or human PAH.
