# Supplementary material for: Metabolism, Mitochondrial Dysfunction, and Redox Homeostasis in Pulmonary Hypertension
Source: Antioxidants (Basel). 2022 Feb 21;11(2):428. doi: 10.3390/antiox11020428 (PMC8869288; doi:10.3390/antiox11020428)
Supplement: Supplementary file 1 [file antioxidants-11-00428-s001.zip › antioxidants-1586872-supplementary.pdf]

Table S1. Summary of key enzymes of different mitochondrial processes that have been associated with animal models of PH and/or human PAH.

Process	Protein/Enzyme	Cell/tissue type	Organism	Model/disease studied	Findings	References
**Glycolytic switch**	α-enolase (ENO1)	N/A<br>N/A<br>PASMCs	Mouse<br>Rat<br>Human	Chronic hypoxia<br>Sugen-hypoxia<br>PAH	-Overexpression of ENO1 associated with apoptosis resistance in PASMC through the AMPK-Akt pathway. Pharmacologic inhibition decreased glycolytic switch	[16]
	G6PD	Cd133+ progenitor	Human	Chronic hypoxia	-Increased G6PD activity associated with upregulation of HIFα	[18]
		N/A	Mouse	Sugen-hypoxia	-Pharmacological inhibition led to decreased in RV pressure	[19]
		N/A	Mouse	PAH	-G6PD-deficient mice had PA and RV remodeling as well as PH. Thought to be related to hemolysis, oxidative stress, and metabolic reprogramming.	[20]
		N/A	Human	PAH	-Select patients with PAH have varying decreased of G6PD deficiency	[21]
	Hexokinase	RV myocytes	Rat	Monocrotaline	-Upregulated, via mRNA and protein expression, in rats with PH.	[21,23]
	Pyruvate dehydrogenase (PD)	Pulmonary artery	Human	PAH	-Increased levels of PDK (inhibitor of PD)	[24]
**Mitochondrial biogenesis**	VDAC & Citrate synthase	PASMCs	Human	PAH	-Lower expression of both VDAC and citrate synthase	[48,49]
	PPARγ	PASMCs	Human	Chronic hypoxia	- Hypoxia leads to decrease expression of PPARγ	[50]
		N/A	Rat	Sugen-hypoxia	- PPARγ agonist decreased RVSP and prevented RV dilation	[51]
**Fission**	DRP1	PASMCs	Human	PAH	-DRP-1 key for cell-cycle checkpoint. Overexpression can lead to hyperproliferation.	[60,61]
		Fibroblasts	Rat	Monocrotaline	-RV fibroblasts had increased expression of DRP1. Inhibition of DRP1 led to decreased proliferation.	[62]

**Fusion**	Mitofusin 2 (MFN2)	PASCMCs	Human	PAH	-SMCs had decreased MFN2 and higher incidence of mitochondrial fragmentation.	[70]
		PASMC	Human Rat	PAH Monocrotaline & Sugen-hypoxia	-Adenoviral-mitofusin 2 overexpression led to decreases of PVR, PA medial thickness and increased lung vascularity.	[70]
**Mitophagy**	UCP2	N/A	Mouse	Chronic hypoxia	-UCP2 knockout (increased mitophagy) mice develop worse hypoxic PH	[95]
**ROS production**	N/A	PA endothelial and SMCs.	Mouse and rat	Chronic hypoxia	-Increased mitochondrial production of superoxide	[107-109]
	Complex I-III	PASMCs	Rat	Monocrotaline	- Lower CI-III activity associated with increased ROS production.	[110]
	NFU1	N/A	Rat	N/A	-Rats with G208C mutation had increased RV pressure, RVH and pulmonary artery remodeling.	[111-113]
	Superoxide dismutase 2 (SOD2)	PASMCs	Human	PAH	-Decreased expression of ETC components and SOD2 resulting in lower ROS production and normoxic activation of HIFα	[115]
	Superoxide dismutase 3 (SOD3)	N/A	Mouse	Chronic hypoxia	-SOD3 whole-body knockout, SMC SOD3 deletion and SNP have been associated with worse hypoxic PH.	[120-122]
